# Is clinician-supported use of a mindfulness smartphone app a feasible treatment for depression? A mixed-methods feasibility study

**DOI:** 10.1016/j.invent.2021.100413

**Published:** 2021-06-05

**Authors:** Clara Strauss, Charlotte Dunkeld, Kate Cavanagh

**Affiliations:** a Sussex Partnership NHS Foundation Trust, Research Department, Sussex Education Centre, Mill View Hospital, Nevill Avenue, Hove, BN3 7HY, UK; b School of Psychology, University of Sussex, Falmer, Brighton, BN1 9QH, UK

**Keywords:** Mindfulness, Headspace, Feasibility study, Depression, Engagement

## Abstract

Depression is the leading cause of disability globally and has serious consequences for the individual, their family and for society. Effective, accessible and affordable treatments are urgently needed. In-person group-based mindfulness-based interventions are an effective treatment for depression, but are not widely available and can be costly. Clinician supported use of mindfulness self-help resources such as mindfulness smartphone applications could widen access at a reduced cost, but there are key feasibility questions that need answering. This is a mixed-methods feasibility study of a blended intervention involving the mindfulness smartphone app Headspace alongside six clinician support sessions with mental health treatment seeking adults experiencing moderate to moderately severe symptoms of depression. In line with recommendations for feasibility studies, we examine whether: (1) it is possible to recruit participants to this novel intervention, (2) participants engage with the intervention, (3) participants and clinicians find the intervention acceptable, and (4) pre-post outcomes on measures of depression (primary outcome), anxiety, wellbeing, mindfulness, self-compassion, rumination and worry indicate effectiveness. Findings show that recruitment is feasible with 54 participants enrolled in the intervention within a 6-month window. In terms of engagement, 44.4% completed at least 80% of recommended Headspace sessions and 72.2% of participants attended at least three clinician support sessions. Clinician-supported Headspace was deemed acceptable by participants and clinicians. Pre-post effect sizes were statistically significant and in the small-medium or medium-large range on all outcomes, with an effect size of *d* = 0.69 (95% CI: 0.34–1.04) for the primary outcome of depression symptom severity. The number of Headspace sessions engaged with was associated with greater reduction in depression symptom severity. Findings suggest that a blended intervention combining Headspace with clinician support has potential as a first-line treatment for moderate/moderately severe depression, but findings are too preliminary to recommend the intervention outside of a research trial. Important caveats are noted including the need for future research to examine predictors of engagement with Headspace sessions so that engagement can be enhanced, to measure the longer term effects of such interventions and to better understand the potential for lasting negative effects of the intervention so that these can be minimised.

## Highlights

• Supported use of mindfulness apps has potential as a treatment for depression.
• This is a feasibility study of Headspace, leading mindfulness app, for depression.
• Recruitment was feasible - 54 participants enrolled within a 6-month window.
• 44.4% completed 80% app sessions or more and 72.2% attended at least 3 sessions.
• Pre-post effect sizes were in the small-medium/medium-large range on all outcomes.

Depression is a leading cause of disability globally and is a major contributor to the global burden of disease (World Health Organization, 2020). It has major personal and economic costs (McCrone et al., 2008), with around 15% of adults experiencing clinically significant depression in any given week (McManus et al., 2009). Major depression is typically recurring, with 50% of individuals relapsing after one depressive episode, and 80% after two (Burcusa and Iacono, 2007). It is not therefore surprising to learn that depression presents a high cost to the economy, including the cost of providing healthcare to people experiencing depression (McCrone et al., 2008). Effective, accessible and affordable treatments for depression are therefore urgently needed.

Self-help cognitive behaviour therapy (CBT) resources supported by a mental health practitioner are recommended in the UK as an affordable first line treatment for depression (National Institue of Health and Care Excellence [NICE], 2011) and are offered in the National Health Service (NHS) in England through its Improving Access to Psychological Therapies (IAPT) service. Whilst supported self-help CBT for depression can be effective, the effect size when compared to usual care is in the small-medium range (Bower et al., 2013) and only 41.0% (computer-based supported self-help) and 40.8% (book-based supported self-help) of people in IAPT with depression achieve recovery following this intervention (NHS Digital, 2019). There is therefore scope to develop alternative self-help treatments for depression to widen patient choice for supported treatments beyond self-help CBT. Widening choice in this way may lead to overall improved outcomes for self-help interventions by matching patients with the treatment most suited to their needs rather than offering self-help CBT to all.

Mindfulness-Based Interventions (MBIs) teach people how to cultivate and apply mindfulness to everyday life. Mindfulness involves paying attention to present-moment experiences in an accepting and non-judgemental manner. Mindfulness-based interventions are traditionally offered in a group setting where participants come together once a week to practice mindfulness and discuss their experiences of home and in-session practice. Randomised Controlled Trials (RCTs) have found that, in comparison to control conditions, MBIs are effective in improving depressive symptomatology (Goldberg et al., 2018; Strauss et al., 2014) and at reducing the risk of relapse for people with a history of recurrent depression (Kuyken et al., 2016). Due to its effectiveness, Mindfulness-Based Cognitive Therapy (MBCT) has been a recommended relapse prevention treatment for depression in the UK since 2004 (National Institute for Health and Care Excellence [NICE], 2009).

There is also increasing interest in learning mindfulness using self-help resources including through books, online programmes and smartphone apps (Cavanagh et al., 2014; Cuijpers et al., 2009; Gu et al., 2018; Jayewardene et al., 2017; Lever Taylor et al., 2014; Victorson et al., 2020), partly driven by limited access to in-person MBI groups in many areas (Crane and Kuyken, 2012; Rycroft-Malone et al., 2019) and by the cost of these courses. Self-help apps such as Headspace (Headspace, n.d.) is becoming increasingly popular, with over 60 million downloads and over 2 million subscribers to date (Headspace, 2020). Meta-analyses of RCTs show that self-help MBIs are effective at improving depressive symptoms in comparison to control conditions (Cavanagh et al., 2014; Spijkerman et al., 2016), however most studies are in non-clinical populations meaning that the effectiveness of self-help MBIs in treating depression in mental health treatment seeking populations is not well understood. In addition, it is unclear if self-help MBIs would be of interest to and an acceptable treatment for people experiencing depression and if a blended approach, integrating self-help MBIs with clinician support, may be warranted.

In comparison to self-help mindfulness books, mindfulness smartphone apps have the advantage of being readily accessible both in and out the home and having built-in mindfulness audio recordings giving easy access to learning mindfulness throughout the day. Whilst there are RCTs showing that Headspace can improve symptoms of depression in non-clinical populations (Bostock and Steptoe, 2013; Fish and Saul, 2019; Flett et al., 2019), there is little known about its potential as a treatment for clinical depression in adults seeking treatment from mental health services. Given the findings that Headspace can reduce symptoms of depression in non-clinical populations it is plausible that these benefits would extend to clinical populations. However, there are some important caveats. First, it is possible that a more intensive and targeted MBI would be necessary to successfully treat depression in clinical contexts, consisting of longer sessions with a specific focus on depression. For instance, MBCT recommends 30–40 min of daily mindfulness practice whereas the standard length of Headspace daily practices is 10 min. Second, a blended approach may be more suitable in a clinical setting whereby an MBI such as Headspace is offered alongside support sessions from a trained clinician. Supportive Accountability theory proposes that guidance in using self-help resources improves engagement and outcomes by increasing accountability (Mohr et al., 2011). In support of this, a randomised controlled trial of internet-delivered MBI for depression with coaching support found significant effects on depression symptom severity in comparison to treatment as usual and a systematic review comparing guided with unguided internet-based (non-mindfulness) interventions found that guided interventions were more effective and had higher rates of engagement than unguided interventions (Baumeister et al., 2014). Finally, it is also not clear if patients seeking treatment for depression would be willing to engage with an MBI smartphone app such as Headspace and/or if they would find the proposed intervention acceptable as a treatment option.

Overall, therefore, clinician-supported use of self-help MBIs delivered by smartphone apps such as Headspace have potential as a treatment for depression but there are a number of feasibility questions that need answering at this early stage in the research journey. This paper presents a feasibility study of supported use of Headspace for people experiencing depressive symptoms accessing in IAPT. In line with recommendations for feasibility studies (Craig, Dieppe, Macintyre, Health, et al., 2008), this mixed-methods study has the following research questions: (1) Is supported use Headspace of interest to treatment seeking adults experiencing symptoms of depression? In other words, can we recruit participants to engage with supported Headspace for depression in IAPT in sufficient numbers to make the treatment sustainable in the service? (2) Can we retain participants in the intervention? That is, do a sufficient number of participants complete a course of supported Headspace to warrant it being a treatment offered in the service? (3) Is supported Headspace acceptable? What do participants and clinicians perceive as the helpful, unhelpful and missing aspects of the intervention? (4) What are the preliminary indicators of effectiveness of supported Headspace? Do pre-post effect sizes on measures of depression (primary outcome), anxiety and wellbeing as well as on measures of purported change mechanisms (mindfulness, self-compassion, worry and rumination) indicate that supported Headspace could be an effective treatment for depression in this population? Findings from this feasibility study will determine whether or not further evaluation of supported Headspace for depression is warranted and, if warranted, what adaptations might be needed.

## 1 Methods

### 1.1 Design

This is an uncontrolled mixed-methods feasibility study following guidance from the Medical Research Council in the UK (Craig, Dieppe, Macintyre, Health, et al., 2008). The aim of a feasibility study is to evaluate whether a novel healthcare intervention is feasible in terms of recruitment, retention and acceptability before proceeding to evaluating effectiveness, if the intervention is deemed to be feasible. As such, evaluating effectiveness is not the primary aim of feasibility studies.

The study received full ethical approval from the London-Surrey Borders NHS Research Committee. The study was pre-registered with ISCRCTN (please see http://www.isrctn.com/ISRCTN13895659).

### 1.2 Participants

Participants were adults referred to an IAPT service based in London, England. Inclusion criteria were as follows, participants were: (1) currently under the care of the IAPT service; (2) had a score of 10–19 (inclusive) on the Patient Health Questionnaire (PHQ-9) – indicating moderate/moderately severe depression symptom severity - at initial assessment; (3) had regular access to a smartphone, computer or tablet with internet access to use Headspace; (4) sufficient literacy skills to read and understand self-help materials; (5) aged 18 or over.

Exclusion criteria were, people: (1) currently receiving another psychological intervention, (2) rated as medium or high risk to self or others on the service risk assessment tool, (3) with substance use associated with significant impairment; (4) meeting diagnostic criteria for post-traumatic stress disorder or obsessive-compulsive disorder (Sheehan, 2014).

### 1.3 Measures

#### 1.3.1 Recruitment

Recruitment was deemed to be feasible if at least 50 participants were recruited to the study over the 6-month recruitment period. This was the number deemed by the service lead to be sufficient to warrant the intervention being feasible to roll out alongside other interventions in the service in terms of resource demands (e.g. time for training and supervision). IAPT practitioners conducting initial assessments were asked to record: (1) the number of eligible people assessed who were offered the study, (2) demographic information (gender, age and ethnicity) for people offered the study, (3) reasons for declining to take part in the study where relevant, and (4) IAPT treatment offered where the study was declined.

#### 1.3.2 Retention

Retention was defined as engaging with at least 80% (24/30) of Headspace Basics Pack practices during the 60-day study period. This is in line with the finding that MBI outcomes for depression are enhanced when participants practice mindfulness at least three times a week (Crane et al., 2014), with 24/30 Headspace practices over the course of the study equating to three practices a week. Retention was additionally defined as the proportion of participants attending at least 50% (3/6) of the Psychological Wellbeing Practitioners (PWP) support sessions. Reasons for not attending at least 50% of PWP sessions were recorded. The number and proportion of participants completing at least 50% (15/30) of practices was also recorded as 50% session attendance is typically taken as the criteria for MBCT treatment completion (Ma and Teasdale, 2004). Headspace usage was automatically collected by Headspace and PWPs recorded session attendance.

#### 1.3.3 Acceptability

##### 1.3.3.1 Qualitative evaluation

Intervention acceptability was assessed through open-ended surveys or interviews with participants, PWPs and service leads.

The first 10 participants agreeing to be interviewed were interviewed using the Change Interview (Elliott et al., 2001). The Change Interview comprises of semi-structured questions which ask participants whether they have noticed any changes in themselves since they began the intervention, in addition to the participants' attribution of these changes (either to the intervention itself or to external, unrelated, factors). Participants were also asked to comment on aspects of the intervention that were helpful and facilitated change, and/or factors that may have been unhelpful or hindered change. The interviews took 30–45 min and were audio recorded to aid transcription and analysis.

PWPs and the service lead were invited to complete an online survey with open-ended questions enquiring about helpful and unhelpful aspects of the intervention, and barriers and enablers to implementing the intervention in the service (service lead only).

##### 1.3.3.2 Intervention Expectation Form (adapted from, Devilly and Borkovec, 2000)

This 6-item questionnaire was administered at Time 1 only to ascertain participants' expectations of the effectiveness of the intervention. It contains both credibility and expectancy scales (3 items per scale). Example items include, “At this point, how logical does the treatment offered to you seem?” (credibility) and “By the end of treatment, how much improvement in your depression do you think will occur?” (expectancy). Participants indicate their responses scales ranging from 1 to 9 on all credibility items. Responses are rated 1–9 on one of the expectancy items and 0–100% on the other two items. The credibility scale produced an alpha of α = 0.84 in this study. As items from the expectancy scale are rated on different scales, responses are first standardised to z-scores. Standardised scores generated an alpha of α = 0.91 in this study.

##### 1.3.3.3 System Usability Scale (Brooke, 2011)

This 10-item measure, adapted specifically for this study, was administered at Time 2 only to assess the usability of the Headspace platform. Example items include, “I found Headspace unnecessarily complex” and “I felt confident using Headspace”. Respondents indicate to what extent they agree with each statement on a Likert scale ranging from 1 (Strongly Agree) to 5 (Strongly Disagree). Reliability analysis demonstrated excellent internal consistency (α = 0.88) in this study.

##### 1.3.3.4 PWP Rating Scale ((adapted Session Rating Scale, Duncan et al., 2003)

This scale includes four items and was administered at Time 2 only to assess the participants' experience of the PWP-led support sessions. An example item includes “Relationship” whereby respondents indicate the extent to which they felt ‘Heard, respected and understood by the practitioner’ (i.e. PWP) on a scale from 1 to 10. Other items focus on goals and topics, approach and method and an overall rating of the support sessions more generally. Duncan et al. (2003) suggest a total score of less than 36/40 suggests that therapeutic support is not optimum and should be explored further. This measure demonstrated excellent internal consistency (α = 0.89) in this study.

##### 1.3.3.5 Lasting Effects Questionnaire (adapted from Crawford et al., 2016)

This questionnaire was administered at Time 2 only to assess any negative effects experienced from the use Headspace. On a scale from 1 to 5, participants are asked to rate to what extent they agree or disagree with the following statement; “I have experienced lasting bad effects from using Headspace”. If respondents are found to agree, they are invited to answer further questions around the specific aspect/s of the intervention that may have contributed to any lasting negative effects.

#### 1.3.4 Effectiveness

Participants completed the following measures at baseline and post-intervention (60-days after baseline).

##### 1.3.4.1 PHQ-9 (Kroenke et al., 2001)

This nine-item self-report tool measures depressive symptom severity and is used in IAPT services across the country. Items are rated on a four-point Likert scale ranging from 0 to 3. Scores 0-4 are conisdered no/minimal depressive symptoms, 5-9 mild levels of depressive symptomatology, 10–14 moderate, 15–19 moderately severe and 20 or over, severe. The scale demonstrated good internal consistency in this study (α = 0.82).

##### 1.3.4.2 GAD-7 (Spitzer et al., 2006)

This seven-item measure looks at the severity of generalised anxiety symptoms. Items are rated on a four-point Likert scale ranging from 0 to 3. Scores ranging between 0 and 4 are considered as no/minimal anxiety symptoms, 5 and 9 are considered mild anxiety symptoms, 10 and 14 moderate anxiety symptoms and 15 or over, severe anxiety symptoms. The scale demonstrated good internal consistency in this study (α = 0.85).

##### 1.3.4.3 Short Warwick Edinburgh Mental Wellbeing Scale (SWEMWBS) (Stewart-Brown et al., 2009)

This measure of mental wellbeing comprises of seven positively-worded statements. Respondents rate how often they have experienced each statement/event over the previous two weeks (e.g. “I've been feeling relaxed”) on a five-point Likert scale ranging from 1 (not at all) to 5 (all the time). A scale total is created and converted using the SWEMWBS Rasch conversion table (Stewart-Brown et al., 2009). The scale demonstrated good internal consistency in this study (α = 0.71).

##### 1.3.4.4 Five Facet Mindfulness Questionnaire (FFMQ-15) (Baer et al., 2006; Gu et al., 2016)

This 15-item measure on mindfulness includes the following five aspects; observing, describing, acting with awareness, non-judging of inner experience and non-reactivity to inner experience. Responses are coded from 1 to 5 to indicate how true each statement is for the individual (e.g. 1 = Never or Rarely True to 5 = Very Often or Always True). The five facets have demonstrated adequate internal consistency in previous literature, ranging from α = 0.69 to α = 0.83 in this study (see Gu et al., 2016) however it is recommended to exclude the Observing subscale in mindfulness naïve populations due to its mixed psychometric properties. In this study a total score for FFMQ-15 minus Observing items is reported in line with recommendations (Gu et al., 2016).

##### 1.3.4.5 Self-Compassion Scale (SCS-SF) (Raes et al., 2011)

The short form version consists of 12 items measuring self-compassion. Responses are coded from 1 to 5 whereby 1 indicates “Almost Never” and 5 indicates “Almost Always”. Example statements include “I try to see my failings as part of the human condition” and “I'm disapproving and judgemental about my own flaws and inadequacies”. This scale demonstrated good internal consistency in this study (α = 0.83).

##### 1.3.4.6 Penn State Worry Questionnaire (PSWQ) (Meyer et al., 1990)

This 16-item measure on worry includes statements such as “I am always worrying about something” and “I find it easy to dismiss worrisome thoughts”. Responses are recorded on a five-point Likert scale whereby 1 represents “Not at all typical of me” and 5 represents “Very typical of me”. Internal consistency analysis was satisfactory for this scale in this study (α = 0.68).

##### 1.3.4.7 Ruminative Responses Scale – Brooding Subscale (Treynor et al., 2003)

The shortened edition of this scale comprises of 10 items and two components: reflection and brooding. Only the brooding subscale is associated with depression vulnerability and this is the scale recommended for use in research. For each item, respondents indicate the frequency of the event on a 4-point Likert scale ranging from 1 (“almost never”) to 4 (“almost always”). Example RRS Brooding items include “Think “Why do I always react this way?”” and “Think about a recent situation, wishing it had gone better”. The Brooding subscale demonstrated adequate internal consistency in this study (α = 0.66).

### 1.4 Intervention

Headspace is an internationally-available digital tool which at the time of the study guided users through a series of non-secular audio-recorded mindfulness practices and animations (since the time of the study additional health and wellbeing content has been added). The content can be accessed via the company's website (www.headspace.com) or by downloading the Smartphone application. Headspace assumes the recipient is not familiar with the concept of mindfulness and therefore directs new subscribers to the 30 session ‘Basics’ packs where foundational aspects of mindfulness are taught through brief mindfulness practices led by one of the company's co-creators. Once completed, a number of other ‘packs’ are available which focus on various wellbeing themes ranging from ‘Happiness’ to ‘Work and Performance’. Headspace informs the user of their progress by letting them know how many sessions they have completed, in addition to how many minutes they have spent meditating. For this study, participants were invited to guide themselves through the 30 session Basics packs and the number of Basics sessions out of 30 completed during the 60-day intervention period was recorded.

The intervention also consisted of six PWP support sessions offered at approximately weekly intervals in order to support participants to guide themselves through the 30-day Basics pack. PWPs are not formal psychological therapists, however they have a year-long training and in-service apprenticeship in delivering low intensity psychological interventions (mostly CBT-based guided self-help). PWP sessions were offered either by phone or in person, depending on participant preference and lasted 30–45 min per session. These support sessions were included in line with Supportive Accountability Theory (Mohr et al., 2011) with the aim of enhancing engagement with Headspace and thereby improving outcomes. Building a trusting therapeutic relationship with the PWP who had expertise in the approach was a crucial part of the intervention with a view to supporting participants to engage meaningfully with the recommended Headspace practices. Support sessions included an exploration of participants' experiences of mindfulness practice using Headspace since the previous session, identifying and addressing barriers to engaging with Headspace practices and developing engagement strategies (where relevant) with a view to enhancing supportive accountability, drawing out participants' learning from mindfulness practice and answering participants' questions about the approach. Each session also included an in-session Headspace practice where the PWP and participant would be guided through the next daily practice together and the PWP would invite the participant to share their experiences of and learning from the practice. The in-session mindfulness practice was either in-person or by phone (depending on the session format). The PWP played the relevant Headspace practice whilst both they and the participant followed the audio guidance. This served the purpose of building a trusting therapeutic relationship (with the participant and PWP practicing mindfulness together) and giving the opportunity to explore in-the-moment experiences of and learning from mindfulness practice and to address any challenges with practice. In line with standard IAPT practice, between-session PWP email or other forms of support was not available.

Six PWPs within the service delivered the intervention. All six received a one-day mindfulness training delivered by the lead author which covered the following topics: (1) what is mindfulness? (2) mindfulness and depression; (3) Headspace Basics pack, and (4) support session structure and process, including opportunities for observation, role play and feedback. As PWPs have a year-long training in how to deliver guided self-help psychological interventions (mostly CBT) for common mental health problems both in person and by phone, the training for the study focused on differences between supporting CBT self-help interventions in person and by phone (which PWPs were already familiar with) and supporting MBI self-help interventions. PWPs were invited to guide themselves through the 30-day Basics Pack as part of their training and were invited to continue to practice mindfulness on a regular basis using Headspace or through other means (e.g. attending local mindfulness groups in the community or using other resources) throughout the study period. PWPs attended a weekly telephone supervision group led by the lead author where participant and PWP experiences of the intervention were discussed, including PWP experiences of their own mindfulness practice both to support PWPs with their own practice and also to model exploration of mindfulness practice.

Technical difficulties with using the Headspace app were addressed by the study research assistant so that PWP support sessions could focus on learning from mindfulness practice.

### 1.5 Procedure

**Fig. 1:**
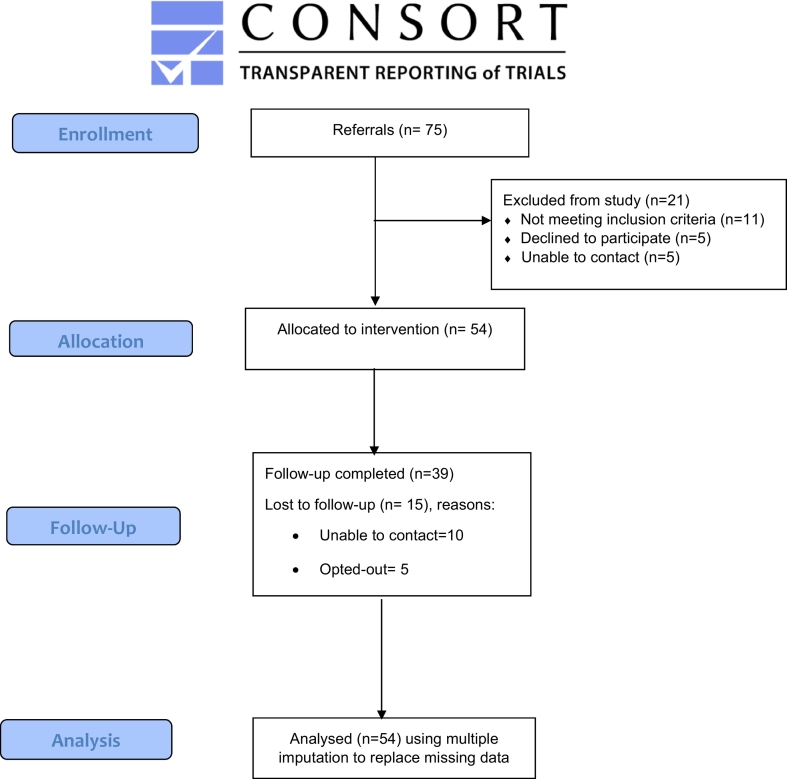
CONSORT Diagram showing participant flow through the study.

Fig. 1 represents the flow of participants through the study. Adults experiencing common mental health difficulties (depression and/or an anxiety condition) can refer themselves into their local IAPT service (or they can ask they general practitioner to refer them). A telephone initial assessment is then conducted by a trained clinician within a few weeks of the referral being received which includes an assessment of current mental health and treatment needs and preferences. Where a common mental health difficulty is identified at initial assessment and where symptoms are mild to moderate/moderately severe a guided self-help intervention (usually CBT) is typically offered. As IAPT is part of the UK's National Health Service, there is no cost for assessment and treatment.

Potential participants meeting the eligibility criteria (above) at initial assessment in the host IAPT service were identified by practitioners and offered the study. The study was offered as an option alongside other interventions offered by the service (typically a range of clinician-support CBT self-help interventions) at the initial assessment appointment, so that all participants had a range of treatment options from which to choose. For people declining to take part in the study, practitioners recorded demographic information, reasons for declining the study and the treatment the person went on to receive in the service. If the person consented to participate, they were sent a Participant Information Sheet, and their contact details were passed onto the researcher who called them to discuss the study in more detail. If the potential participant was happy to proceed, a further telephone call was arranged with the researcher to electronically sign the consent form and to conduct the baseline assessment. After the baseline assessment was completed, the researcher set the participant up with their Headspace subscription and booked in their initial PWP support session. Participants were advised not to access Headspace until the initial support session with their PWP. Support sessions were held either in person at the IAPT service or over the telephone, depending on participant preference. Sixty days after the first PWP session the researcher contacted the first 10 consenting participants by telephone to carry out the Change Interview. All participants were sent the post-intervention measures. At the end of the study, the PWPs and the IAPT service leads were sent the anonymous feedback questionnaire.

### 1.6 Data analysis plan

Data were analysed using SPSS version 25. Recruitment and retention data are reported descriptively and unimputed means and standard deviations on outcome measures at baseline and post-intervention are reported. Multiple imputation was used for baseline to post-intervention comparisons using paired *t*-tests to take account for missing data at post-intervention. Multiple imputation was carried out for outcomes with more than 5% missing data under the assumption that the data were missing at random (Jakobsen et al., 2017; Sterne et al., 2009) with 30 datasets imputed and results pooled. Multiple imputation for chained equations (White et al., 2011) was used and included any variables associated with outcome data missingness or that were correlated with post-intervention outcomes. Outcomes that correlated with missingness were identified using Missing Value Analysis in SPSS with statistical with significance set at the 10% level to be less conservative with the variables to include in the multiple imputation model given the size of the data set (Fisher, 1992). Outcomes with less than 5% missing data were automatically subject to a complete case analysis; participants with missing data for the outcome being analysed were excluded from its analysis. The standardised effect size for each pre-post comparison was calculated using the imputed data as Cohen's *d* with 95% confidence intervals for *d* reported given. Cohen's d can be interpreted as: 0.2 = small, 0.5 = medium, 0.8 = large. Analyses were conducted for both the intention-to-treat sample (primary analysis) and the per protocol sample (including only those participants who completed the intervention).

Qualitative data from the Change Interview was transcribed and analysed using Thematic Analysis (Braun and Clarke, 2006). Audio recorded interviews were transcribed verbatim and transcripts were organised into discrete codes. Two researchers organised codes into themes and subthemes with this process being supervised by the first author. Disagreements with this process were resolved by discussion between the two researchers and the first author. Pseudonyms are used for extracts from interviews in order to ensure anonymity.

## 2 Results

### 2.1 Recruitment

A total of 95 people were offered the study by a clinician following their initial assessment in the IAPT service, of whom 75 expressed an interest in and were referred into the study and of whom 54 were eligible and consented (72% of those referred). Following referral, reasons for not consenting were: not meeting inclusion criteria (n = 11), declining to participate (n = 5) and inability to contact (n = 5). This means that the recruitment target for the study of recruiting at least 50 participants within six months was met.

There were a variety of reasons for people declining to take part in the study. Reasons for declining included preference for a specific type of treatment (e.g. counselling), previous use of Headspace and a wish to try an alternative and doubt that a smartphone app would be helpful.

The demographic profiles of consenting participants can be found in Table 1. A total of 21 people who were referred did not take part in the study. Where demographic data was available for people not taking part in the study, 9 were female, 9 were male, the mean age was 36 years (range 19–64 years) and 6 were of Black, Asian or Mixed ethnicity and 7 were White.

**Table 1:** Demographic profile of consenting participants (n = 54).

Variable	Frequency/n	Percentage of sample/%
Gender	Female (37)	68.5%
	Male (17)	31.5%
	Other (0)	0%
Age	Mean = 36 years	
	Range: 20 to 59	
Ethnicity	Asian (11)	20.4%
	Black (10)	18.5%
	Mixed/multiple ethnicity (7)	13.0%
	White (24)	44.4%
	Other (2)	3.7%

### 2.2 Retention

Participants attended a mean of 4.11 (SD = 2.21) PWP support sessions with 39/54 (72.2%) participants attending at least 3 support sessions. Participants completed a mean of 19.54 (SD = 11.06) of the 30 Headspace Basics pack practices within the 60-day intervention period. A total of 24/54 (44.4%) participants completed at least 24 of the 30 guided mindfulness practices from Headspace's Basics Packs, within the 60-day intervention period (34/54 (63%) participants completed at least 15 of the 30 Basics pack practices and 17/54 (31%) participants completed all 30 Basics pack sessions).

In total, 23/54 (42.6%) participants completed the supported Headspace intervention as defined in this study – that is, completing at least 24 of the 30 Headspace Basics pack practices and attending at least 3 PWP sessions within the 60-day intervention period.

Reasons for not attending at least 3 PWP sessions were: lack of time to attend PWP sessions (n = 5), lack of time to engage with mindfulness practice (n = 3) and not finding the intervention helpful (n = 2). Reasons were not given by 5 people as it was not possible to contact them.

### 2.3 Acceptability

#### 2.3.1 Participant session ratings

Participants rated the PWP sessions a mean of 33.84 (SD = 7.62) out of a possible 40 on the adapted PWP rating scale. Scores ranged from 15 to 40. Duncan et al. (2003) note that scores under 36 could be a cause for concern and may indicate that the therapeutic relationship could be enhanced. A total of 58% of participants gave the PWP sessions a rating of 36 or higher meaning that 42% of participants rated sessions below the 36 threshold.

#### 2.3.2 Headspace usability

The mean system usability score in our sample was 80.14 (SD = 20.66, range 12.50–100) out of a possible 100, which suggests excellent system usability (i.e. most participants found Headspace to be a very accessible platform).

#### 2.3.3 Intervention credibility and expectation

The mean score on the credibility subscale of the Credibility and Expectancy Questionnaire (Devilly and Borkovec, 2000) was 6.26 (SD = 1.40) out of a maximum possible score of 9. The expectancy subscale items are rated on different scales so these are reported by item. The item means/maximum possible score (and standard deviations) for the three items were: 53.70/100 (21.22), 5.83/9 (1.59) and 52.59/100 (21.99) respectively. The expectancy and credibility scales demonstrated significant positive correlations with the number of Headspace Basics Pack sessions completed; (*r* = 0.32 *p* = .02; *r* = 0.30 *p* = .03, respectively), showing that more Headspace sessions were completed where treatment expectancy and credibility were higher at baseline. However, correlations between these subscales and pre-post improvements on all outcome measures were non-significant when having *p* < .01 to take account of multiple tests.

### 2.4 Participant experiences of the intervention

Thematic Analysis was conducted on transcribed interview data to examine participants' experiences of the intervention. Deductive thematic analysis was conducted whereby each of the broad interview questions formed the basis for overarching themes: helpful/engaging aspects, unhelpful aspects/barriers and lasting positive/negative effects.

#### 2.4.1 Helpful and engaging aspects of the intervention

##### 2.4.1.1 Subtheme 1: PWP support

One prominent theme to emerge for the analysis was the importance of the PWP support sessions to facilitate engagement with Headspace. For example, the majority of participants discussed how the support sessions provided them with motivation, reassurance and encouragement, especially if, due to low mood, they were struggling to incentivize themselves:

*I think for some people it would be really helpful to have an additional person to be able to talk to them about it. Erm, I think coz partly, I don't know, if you are feeling emotionally drained, it could be kind of hard to keep on motivating yourself to keep on doing it, or it could be, as I say, just another thing to have on the to-do list, erm so having a supportive person alongside who just encourages, talks you through it, is definitely helpful.*

*(Paul)*

##### 2.4.1.2 Subtheme 2: accessibility of the intervention

The accessibility of having Headspace immediately available on a smartphone was highlighted by some participants:

*And then I was just, well away. Coz I hadn't gotta go turn the laptop on and do this and do that, it didn't become a thing.*

*(Elsie)*

as was having the option of PWP telephone support sessions so as not to have the inconvenience of travel:

*I guess my other point was that it was really good to be advertised for people like me who can't necessarily go to someone face-to-face during the week, but also for people with full-time jobs, it's really hard for them to do something like that.*

*(Lucy)*

##### 2.4.1.3 Subtheme 3: acquiring new skills and attitudes

Another subtheme concerned the benefits of learning new skills:

*And then you can educate someone to help themselves if it happens again. Because this is like CBT in the way that it gives you tools… to help yourself.*

*(Elsie)*

Increasing awareness (of the environment, of thoughts, of choices) and acceptance of thoughts were skills mentioned by several participants:

*Erm, which really helps clear my mind and centre me. Um, or two I have sometimes, erm, uh, kind of like, focused on the sounds around me for a minute or two. Erm, and just kind of picked them out and noticed them.*

*(Arthur)*

*So yeah, it was more like, awareness of when, you know, your mind is racing and, it, you know, just gives you an opportunity to step back and sort of view… yeah, sort of, take a non-biased approach to how you are thinking.*

*(Steve)*

Whilst Megan reflected on mindfulness bringing awareness to choices:

*So I feel, in that essence, quite a lot of peace. Um, yeah, so there's that. I think the other main thing was realising, feeling quite empowered, realising that that I have a choice. Um, in that looking at my, in becoming aware, it highlights the choices that I have.*

*(Megan)*

##### 2.4.1.4 Subtheme 4: regular practice

Making mindfulness practice part of a daily routine was highlighted by some participants, for example, Megan stated:

*But I think, I think getting into a bit of a routine, I surprised myself as I didn't think I would be as regular with it as I have been.*

*(Megan)*

#### 2.4.2 Unhelpful aspects or barriers to engagement

##### 2.4.2.1 Subtheme 1: limited support and tools

A salient theme for some of the participants was that the intervention did not provide sufficient support or sufficient tools to fully address their difficulties. Despite reporting some benefits, one person felt as though their lack of motivation had not been addressed; the other wanted more of a personalised approach to discuss specific experiences:

*Participant: Yes, well that's my biggest thing. That back to work, and I still feel not ready for that, and although I've got kind of, the breathing exercises that I could do, I don't feel like it's enough at the moment.*

*Researcher: Yeah.*

*Participant: So, I just need to work on that somehow.*

*(Sophie)*

*Participant: I think the difficulty I had is that work got a bit too, it got rather busy, there are a few other sort of issues, with my girlfriend, my family that added all sort of stresses, so it became harder and harder to find time to dedicate to practices. And it's, it was more that sort of thing that I felt, it then wasn't actually, I guess, addressing some of the things I wanted to talk about, which I guess was some of the stresses in my life … So I guess I wanted to talk a little more about that rather than just, just focusing on mindfulness.*

*(Steve)*

##### 2.4.2.2 Subtheme 2: mindfulness practice as a challenge

Participants discussed some of the difficulties with mindfulness practice, such as feelings of boredom and frustration with mind-wandering and feeling somewhat driven to practice so that it could feel prescriptive and akin to a chore:

*It could get, it could get quite, I wouldn't say boring, but quite, erm, monotonous, mundane, to just still be constantly doing the same thing and not to be thinking or focusing on anything.*

*(Lucy)*

*Participant: Erm… I think… I think, I think, I think to be honest the fact that I was quite um, hard on myself, um, when I started to not find the time to practice as much. It was a very negative thing in terms of how I viewed myself.*

*Researcher: Hm*

*Participant: Erm, and that sort of perversely, incentivised me to sort of, practice more, because then Headspace become sort of, associated with, sort of, a stressful thing for me.*

*(Steve)*

A couple of interviewees found the decrease in verbal guidance as users progress through Headspace's Basics packs unhelpful:

*Participant: Yeah, in the later sessions where er, there are long pauses between when Andy's saying something and you are supposed to be doing that, so for example if it was breathing and counting to whatever, there's like, a long silence, the silences get longer.*

*Researcher: Hm*

*Participant: And my mind would wander quite a lot, which is fine but then I would be getting frustrated with it.*

*(Sophie)*

##### 2.4.2.3 Subtheme 3: practical barriers to mindfulness practice

Some participants acknowledged finding it difficult to make time and find a suitable environment to practice:

*No, that was the main factor, but I did feel a bit silly. It was better at home and then I tried various different ways to do it, and I think at first, especially I think with someone else in the room, it felt weird.*

*(Kathryn)*

*Like the journey home was too, is too busy, so I'd usually do it when I got home, so I just went through a period where I'd get home a bit later and then, you know, there would be sort of, calls from family and other things that I needed to deal with, and that took up my time, and then I still needed to do all the rest of the, you know, IAPT admin, and I, you know, it just became a bit more difficult to find the time I guess.*

*(Steve)*

One participant observed technology as a potential barrier:

*The only thing is, I'm not IT savvy, I'll be honest, I am a bit, I've been dragged into the 21st Century, but if I didn't have the app on my phone I would get a new phone but then I would get my husband to put it on for me, but, that's the only thing, if you're not IT savvy I suppose. I mean, you make it simple enough, I mean, I think obviously you are going to get people, I suppose older people maybe, who may be struggling.*

*(Paula)*

#### 2.4.3 Positive and negative effects of the intervention

Some of the short-term positive effects to emerge from the interviews were; improved sleep, enhanced clarity of thought, greater self-compassion, feeling calm, reduced worry and catastrophization, greater resilience to stress and improved social relationships. No lasting negative effects were reported in the qualitative interviews. One interviewee did state that becoming more ‘aware’ of their symptoms at the beginning was challenging, and initially exacerbated their symptoms, as quoted below:

*Because of that I did find the first few sessions quite difficult and probably, sort of, symptom-wise, got a little bit worse because I was becoming more aware.*

*(Megan)*

However, they were able to continue with the intervention and reported benefits by the end of the study.

### 2.5 Psychological Wellbeing Practitioner (PWP) feedback

Four of the six PWPs who delivered support sessions completed the PWP survey. Overall, they found it to be a positive experience, but only for certain clients.

#### 2.5.1 Helpful aspects of the intervention

There were aspects of the intervention that PWPs found to be particularly helpful and engaging. They reported that it helped participants to establish links between their thoughts and feelings, enabling them to create ‘space’ between these aspects of experience. They found that the intervention provided participants with protected time for daily for self-care. They reported that participants found Headspace user-friendly, appreciated how accessible it was and valued the self-help element. A few of the PWPs stated that the acceptance and non-judgmental components of the intervention were especially poignant for some of the participants, and that most participants appreciated the support sessions alongside access to the Headspace platform. One PWP remarked that the delivery of the intervention felt quite ‘containing’ for them, as there was less of a focus on the content of client thoughts.

#### 2.5.2 Unhelpful aspects of the intervention

PWPs also commented on unhelpful aspects of the intervention, as well as potential barriers to engagement. One PWP, for example, observed how a client with physical health problems found the intervention to be unaccommodating, which impacted upon their level of engagement. This participant that the Headspace app did not provide enough information/support on the application of mindfulness to chronic physical health problems. Other barriers to engagement suggested by PWPs were the participants' level of concentration and their overall understanding of the mindfulness approach. As with a couple of the participant interviews (above), PWPs also recognised that the intervention was not able to provide some participants with sufficient support or skills. One of the PWPs acknowledged that participants could find the application challenging to navigate due to the numerous ‘packs’ on offer. Several participants also experienced technical difficulties which led to frustration. One PWP commented on how participants could find the process repetitive and the PWP support sessions too long.

### 2.6 Service lead feedback

The IAPT service lead reflected that, as with all changes within IAPT services, the intervention was somewhat challenging to implement as it required additional staff training and supervision, changes to client care pathways and online systems, and time to educate staff members on a new form of treatment. The service lead considered strong evidence for the mindfulness-base cognitive therapy (MBCT) to be one enabler to implementing the intervention into the service more fully as this gave the current intervention more credibility. Another enabler was understanding the rationale for PWP support over and above offering Headspace as a standalone intervention.

Barriers to implementation included the additional demand on PWP time for training and supervision. The service lead also mentioned lack of therapist buy-in to be another potential barrier, as a few of the PWPs facilitating the intervention were not always convinced of the benefits themselves.

### 2.7 Lasting Effects Questionnaire

A total of 3 participants of the 35 who answered the question (9%) reported that they either strongly or slightly agreed with the statement “I have experienced lasting bad effects from using Headspace”. These participants were asked for further information about lasting negative effects, but no information was given. All three participants showed pre-post intervention improvement in depression symptom severity (PHQ-9).

### 2.8 Preliminary indicators of effectiveness

To assess preliminary indication of effectiveness, pre- to post-intervention Cohen's d effect sizes and 95% confidence intervals (CIs) were calculated for all participants using imputed data alongside *t*-tests of pre-post changes. For details of statistical test findings please refer to Table 2.

**Table 2:** Pre-post effect sizes (*d*) and *t*-test findings for intention-to-treat sample with multiple imputation of missing post-intervention data (n = 54). Means and standard deviations are given for the original (unimputed) data (n = 39).

Variable	Pre-treatment M (SD)	Post-treatment M (SD)	Pre-post *d* (95% CI)	*t* (*p*)
PHQ-9	11.24	6.26	0.69	4.13 (<0.001)
	(5.22)	(4.73)	(0.34–1.04)	
GAD-7	10.08	5.42	0.69	4.08
	(4.86)	(4.58)	(0.33–1.05)	(<0.001)
SWEMWBS	18.82	21.13	0.42	−2.65
	(2.50)	(4.97)	(0.10–0.74)	(0.009)
RRS Brooding	13.42	11.89	0.34	2.19
	(4.91)	(2.87)	(0.03–0.66)	(0.031)
SCS-SF	29.84	35.87	0.48	−3.32
	(8.53)	(8.34)	(0.88–0.78)	(0.001)
PSWQ	61.67	54.33	0.36	2.81
	(9.49)	(11.24)	(0.10–0.63)	(0.006)
FFMQ-O	36.28	40.74	0.43	−2.64
	(6.56)	(6.82)	(0.10–0.76)	(0.01)

#### 2.8.1 Depression, anxiety and wellbeing

Pre- to post-intervention effect sizes were medium-large and statistically significant for depression symptom severity (PHQ-9) – the primary outcome - and anxiety symptom severity (GAD-7). For wellbeing (SWEMWBS) the pre-post effect size was in the small-medium range and statistically significant.

#### 2.8.2 Proposed mechanisms of action

Pre-post improvements for proposed mechanisms of action were statistically significant and in the small-medium range for mindfulness (FFMQ-15 minus Observe), self-compassion (SCS-SF), worry (PSWQ) and rumination (RRS Brooding).

##### 2.8.2.1 Per-protocol analysis

Findings were replicated for the intervention per-protocol sample (the n = 23 participants who completed at least 24 of the 30 Headspace Basics pack practices and attended at least 3 PWP sessions within the 60-day intervention period), although effect sizes were mostly numerically larger. For depression (PHQ-9), the primary outcome, the pre-post effect size was numerically larger at *d* = 1.12 in the per-protocol sample. The pre-post change in rumination (RRS brooding) just failed to meet statistical significance, possibly due to the reduce sample size for this analysis. Full findings for the per-protocol sample can be found in Table 3.

**Table 3:** Pre-post effect sizes (*d*) and t-test findings for per-protocol sample using multiple imputation for missing post-intervention data (n = 23). Means and standard deviations are for original (unimputed) data (n = 21).

Variable (N)	Pre-treatment M (SD)	Post-treatment M (SD)	Pre-Post *d* (95% CI)	*t* (*p*)
PHQ-9	13.30	6.75	1.12	4.24
	(4.90)	(5.41)	(0.50–1.73)	(<0.001)
GAD-7	13.20	6.75	1.18	4.46
	(2.33)	(5.20)	(0.56–1.81)	(<0.001)
SWEMWBS	18.04	20.62	0.58	−2.79
	(2.13)	(3.74)	(0.14–1.03)	(0.005)
RRS brooding	14.05	12.37	0.48	1.85
	(2.66)	(2.81)	(−0.05–1.01)	(0.065)
SCS-SF	28.35	33.75	0.48	−2.42
	(8.86)	(9.26)	(0.07–0.90)	(0.016)
PSWQ	67.89	59.26	0.65	3.32
	(6.60)	(10.35)	(0.22–1.08)	(0.001)
FFMQ-O	34.81	40.00	0.63	−2.67
	(5.80)	(6.95)	(0.13–1.13)	(0.008)

##### 2.8.2.2 Recovery rates

In IAPT services recovery is deemed to have occurred where a person has moved from scoring above the clinical cut-off on the PHQ-9 (>9) and/or the GAD-7 (>7) at their initial IAPT assessment to below clinical cut-offs on both measures at post-intervention. Using these criteria, 44.4% of participants in the current study recovered following the clinician-supported Headspace intervention.

##### 2.8.2.3 Relationship between intervention engagement and outcome

Pre-post improvement in depression score (PHQ-9) using imputed data was correlated with the number of Headspace Basics sessions completed (*r* = 0.38, *p* = .005), with a medium effect size, showing that completing more Headspace sessions was associated with greater improvement in depression symptom severity. Pre-post improvement in depression score however was not correlated with the number of PWP sessions attended (*r* = 0.14, *p* = .32).

## 3 Discussion

The aim of this mixed-methods study was to investigate the feasibility of a blended intervention involving a mindfulness smartphone app (Headspace) alongside support sessions from a trained clinician for treatment seeking adults experiencing symptoms of depression. In line with recommendations for feasibility studies (Craig, Dieppe, Macintyre, Michie, et al., 2008), feasibility was assessed through examining recruitment and retention rates, participants' and clinicians' experiences of intervention and pre-post intervention effect sizes on primary (depression) and other outcomes using both quantitative and qualitative methods.

### 3.1 Recruitment

Recruitment was feasible. Over half of people offered the study consented to participate and the target of recruiting at least 50 participants within the 6-month recruitment period was met. Reasons for declining the study included potential participants wanting a non-MBI intervention and not wanting to take part in a research study. The latter reason is in relation to the research study, rather than to the intervention and therefore would likely not be relevant if this intervention is shown to be effective and is offered as part of routine care. The numbers recruited (n = 54) suggests that the intervention would be feasible to offer in an IAPT service given the resources required for training and supervising PWPs.

### 3.2 Retention

Almost three-quarters (72.2%) of participants attended at least 3 PWP support sessions with participants attending 4.11 sessions on average. This is comparable to supported self-help CBT for depression in IAPT where, in the most recent year for which data are available, participants attended an average of 4.3 and 4.0 sessions for self-help book and computerised self-help respectively (Health and Social Care Information Centre (HSCIC), 2019). In terms of engagement with the Headspace sessions, almost half of participants (44.4%) completed at least 80% of Basics Pack sessions within the 60-day intervention period and 63.0% completed at least half of the Headspace Basics Pack sessions in this timeframe. On average, participants completed 19.54 Headspace practice sessions over the 60-day intervention period (SD = 11.06) which equates to 3 h and 15 min. This compares favourably to engagement with CBT-based smartphone apps for depression where the median duration of app use over the 8-week intervention period was 3.0 h in one study (IQR 1.7–5.0) (Zhang et al., 2019). This suggests that engagement with smartphone apps for depression, whether they are based on mindfulness or CBT principles, may be similar, although a direct head-to-head comparison is needed. It is also interesting to note the large between-participant variability in engagement in both studies (i.e. SD = 11.06 in the current study and IQR = 1.7 to 5.0 in Zhang et al., 2019). Understanding predictors of engagement with these apps is a crucial question for future research, particularly as completing more Headspace practice sessions was associated with a greater reduction in depressive symptomatology suggesting that strategies to improve engagement with the app could improve outcomes.

### 3.3 Acceptability

The interview feedback provided by ten study participants was generally positive; all participants mentioned experiencing benefits from the intervention. Subthemes that emerged from the thematic analysis were: the importance of PWP support, accessibility of the intervention (both of the Headspace app and of PWP support), acquiring new skills and attitudes through mindfulness, establishing a regular mindfulness practice, limited support and tools, challenges of mindfulness practice and practical barriers to mindfulness practice.

The majority of participants emphasised the importance of the PWP support sessions to provide encouragement and promote engagement with Headspace sessions. This is supported by studies suggesting that guided MBIs appear to have improved outcomes compared to unguided MBIs (Allexandre et al., 2016) and by meta-analysis of RCTs showing that clinician-supported CBT self-help for depression (Richards and Richardson, 2012) and guided internet-based mental health interventions (Baumeister et al., 2014) are more effective and have lower attrition than unsupported self-help. Therefore, whilst unsupported use of Headspace for depression might be a more efficient use of resources, it may be less clinically effective than supported use of Headspace and ultimately less cost effective. These findings are consistent with Supportive Accountability Theory which suggests that guidance in using self-help resources is important because it improves engagement and outcomes by increasing accountability (Mohr et al., 2011). Moreover, there are concerns that, in keeping with other psychological approaches, mindfulness practice could be unhelpful or even harmful for some people (Baer et al., 2019). Clinician support is one important way to monitor and manage potential challenges and harmful effects at an early stage.

There were a range of experiences of mindfulness practice using the Headspace app. For some, mindfulness practice led to acquiring new, non-judgemental attitudes towards experience and the realisation of choices that are available. Other participants found practice to be mundane or struggled with long periods of silence, whilst others noted challenges with finding the space and time to practice or technological challenges with accessing Headspace. This range of experiences with mindfulness practice in MBIs is not unusual (Banerjee et al., 2017) and mindfulness practice is of course not intended to be easy or blissful. Rather, mindfulness involves paying curious, non-judgemental attention to whatever is present, allowing experiences to be just as they are. If boredom or frustration is noticed, that is simply what is present in the moment and gives the opportunity to practice kind, non-judgemental awareness of unpleasant experiences. A skillful mindfulness teacher can embody and support such an attitude towards experience and, in the case of the current intervention, this is a role both for the Headspace teacher (Andy Puddicombe) and the PWP. The range of experiences of mindfulness practice in this study again highlights the important role the PWP can play in embodying kind, non-judgemental awareness and openness to experience whatever arises.

PWPs delivering the intervention were generally positive about the approach, but highlighted that it may not be suitable for all. In particular, they highlighted that the intervention may be less suitable for those experiencing chronic health conditions or more severe mental health difficulties. The majority of participants (58%) rated their PWP sessions above the session rating threshold on the adapted PWP rating scale. The leaves some room for improvement and highlights the potential need for refining the PWP training and supervision. It could reflect the fact that PWPs do not have a formal psychological therapy training and accreditation and that the 9/10 per-item threshold recommended by Duncan et al. (2003) is unrealistic for a non-therapist PWP workforce, however, refining training and supervision may enhance participant ratings of PWP sessions. Session ratings might be enhanced by adopting the Efficiency Model of Support (Schueller et al., 2017) which suggests that failure to benefit from behavioural intervention technologies can occur for five reasons: (1) usability, (2) engagement, (3) fit, (4) knowledge, and (5) implementation. Training of PWPs could focus on how best to support participants within these five areas (e.g. using direct and indirect communication and information to guide sessions, such as Headspace usage data). Findings from the System Usability Scale suggest that many participants found the design of the Headspace app particularly attractive and easy to use. This is promising given that research has found app usability to be associated with engagement (Enrique et al., 2019).

Nine percent of participants responded that they either strongly or slightly agreed with the statement ‘I have experienced lasting bad effects from using Headspace’. Further details of the lasting effects were not given although all three participants reporting lasting negative effects showed pre-post intervention improvements in depression symptom severity. There are negative effects of psychological treatments more broadly with a recent large-scale study showing 5.2% of respondents reported lasting negative effects following psychological treatment for common mental health problems (Crawford et al., 2016). Therefore, it is not unexpected that a small proportion of participants in the current study reported lasting negative effects. However, it is imperative to minimise the risk of lasting negative effects and future research should attend to reasons for these as well as identifying if there are groups of people more likely to experience lasting negative effects. Findings from such research can be used to minimise the risk of lasting negative effects by adapting the intervention and/or by establishing intervention suitability criteria.

### 3.4 Preliminary indicators of effectiveness

Pre-post improvement on the primary outcome, depression symptom severity, was in the medium-large range (*d* = 0.69). This is somewhat smaller when compared to findings from a recent RCT of internet-delivered CBT for depression (Richards et al., 2015) which found a pre-post effect size of *d* = 0.91 on depression symptom severity, although it is not possible to directly compare without a head-to-head comparison. Overall, 44.4% of participants in our sample recovered following the intervention which compares favourably with the 41.0% (computer-based supported self-help) and 40.8% (book-based supported self-help) recovery rates for depression in IAPT (NHS Digital, 2019). Pre-post improvements on secondary clinical outcomes of anxiety and wellbeing were in the medium-large and small-medium range respectively. In terms of proposed mechanisms of action, pre-post improvements were found for mindfulness, self-compassion, worry and rumination in the small-medium range.

Whilst uncontrolled, these findings are encouraging as they suggest that clinician-supported use of Headspace has the potential to be an acceptable and effective intervention for moderate/moderately severe depression. Pre-post improvements on proposed mechanisms of mindfulness, self-compassion, worry and rumination are consistent with these being mechanisms of change in line with research examining mechanisms of in-person MBI groups (Gu et al., 2015). However, there is scope to adapt the clinician-supported intervention to improve engagement and enhance effectiveness.

### 3.5 Strengths and limitations

One strength of the study was its ecological validity. The intervention was implemented in a real-world mental health service with clinicians from the service delivering the intervention to adults seeking treatment from the service. In order to gauge feasibility as accurately as possible, the study was designed to mirror real-world implementation. Another strength of the study was the use of a mixed-methods approach, giving a richer and more comprehensive analysis of intervention feasibility.

There were also a number of limitations with the study. First, there was data missing at post-intervention despite efforts made to retain participants in the study, although multiple imputation was used to take account of missing data. Future research should employ additional methods to retain study participants including use of payments for completion of assessments. Second, this was a feasibility study which meant that the design was uncontrolled. Whilst recommended for a feasibility study where gauging effectiveness is not the primary aim, future research should examine effectiveness within an RCT design. Third, maintenance of effects was not explored. Future research should include longer-term follow-up. Fourth, PWPs did not have access to Headspace usage data on a weekly basis as this was acquired from Headspace at the end of the study. Instead, PWPs relied on participants self-reporting Headspace usage. In future research, live Headspace usage reports could be helpful for PWPs to identify and address barriers to engagement as it is possible that participants' self-report of usage may not always be accurate (e.g. feeling embarrassed to acknowledge lack of engagement to the PWP). Finally, Headspace usage data records a mindfulness session as complete when a session is started, regardless of whether or not the session is completed. This means that it is not possible to distinguish between mindfulness sessions that were completed in full and sessions that may have been ended prematurely by a participant. In future, it would be useful if actual session time was recorded to allow for fuller exploration of engagement and of the relationship between engagement and outcome.

### 3.6 Implications

This is an early-stage feasibility study and as such it is too early to recommend clinician-supported use of Headspace as an intervention for depression based on our findings. Findings however suggest that clinician-supported use of Headspace as a first-line treatment for depression has potential and should be explored in future research studies. In future research, we suggest three important considerations should be taken into account. First, participant feedback suggests that PWP support was an integral part of the intervention and contributed to its benefits, suggesting that offering Headspace as a standalone treatment for depression may not be suitable. Future research into supported mindfulness-based self-help interventions in clinical contexts should include further development and implementation of a written training curriculum and supervision guidelines for clinicians supporting the intervention to ensure optimisation of practitioner learning and patient experience. Second, as with CBT-based apps for depression, engagement with the 30 Headspace Basics Pack sessions was variable. Given the association between pre-post improvement in depressive symptom severity and engaging with Headspace practice sessions, further research is needed to understand this variability. That is, what helps and hinders engagement with the Headspace sessions? Research testing models of health behaviour such as the Theory of Planned Behaviour (TPB) (Ajzen, 1991) are warranted to improve understanding of engagement in self-help MBIs such as Headspace. For example, the TPB suggests that a health behaviour (if we conceptualise mindfulness practice as a health behaviour given its association with good mental health) is predicted by the intention to engage in the behaviour, which in turn is predicted by attitudes towards the behaviour, subjective norms and perceived behavioural control. By testing such a model we can hopefully better understand factors associated with engagement in Headspace and develop approaches to target these factors and potentially improve engagement and associated outcomes. Third, it is of note that three participants reported lasting negative effects of the intervention. Further research is needed to understand the nature of these lasting effects and what could be done to minimise the risk of such effects occurring. Finally, the mean PHQ-9 score at baseline was 11.24 showing that, on average, participants were scoring in the moderate (10–14) rather than moderately severe (15–19) range. Findings therefore may reflect participants experiencing moderate rather than moderately severe symptoms of depression and this should be taken into account when designing future research studies (i.e. exploring the potential of the intervention for people experiencing moderately severe symptoms of depression). It may also reflect the fact that clinicians were reluctant to refer people into the study who were scoring in the moderately severe range and exploring clinicians' reasons for referring and not referring would be worthy of investigation in future research.

### 3.7 Conclusions

This mixed-methods feasibility study of a blended intervention for depression involving Headspace alongside clinician support suggests that it is feasible in terms of ability to recruit participants to the intervention. In terms of retention, whilst more than 7 out of 10 engaged with at least 3 PWP sessions, less than half engaged with 24 or more of the 30 Headspace Basics Pack sessions recommended during the intervention period. We suggest future research focuses on better understanding the factors that help and hinder engagement with Headspace sessions. The intervention was deemed acceptable, with clinician support being highlighted as integral to good outcomes, suggesting that the blended nature of the intervention (Headspace alongside clinician support) was key. Pre-post improvements on depressive symptom severity were medium-large and suggest promise for this intervention. Further research is now needed to enhance clinician support and to improve rates of engagement with Headspace sessions.

## Funding

The study was funded by Headspace (www.headspace.com).

## Declaration of competing interest

CS is the joint lead for Sussex Mindfulness Centre and has been Chief Investigator on NIHR (UK) and other grants evaluating mindfulness-based interventions. KC has received research funding to evaluate mindfulness-based interventions. Headspace funded the study but had no involvement in data collection or data analysis or in approving the final manuscript.

## References

[bb0005] Ajzen I. (1991). The theory of planned behavior. Organ. Behav. Hum. Decis. Process..

[bb0010] Allexandre D., Bernstein A.M., Walker E., Hunter J., Roizen M.F., Morledge T.J. (2016). A web-based mindfulness stress management program in a corporate call center. J. Occup. Environ. Med..

[bb0015] Baer R., Crane C., Miller E., Kuyken W. (2019). Doing no harm in mindfulness-based programs: conceptual issues and empirical findings. Clin. Psychol. Rev..

[bb0020] Baer R.A., Smith G.T., Hopkins J., Krietemeyer J., Toney L. (2006). Using self-report assessment methods to explore facets of mindfulness. Assessment.

[bb0025] Banerjee M., Cavanagh K., Strauss C. (2017). A qualitative study with healthcare staff exploring the facilitators and barriers to engaging in a self-help mindfulness-based intervention. Mindfulness.

[bb0030] Baumeister H., Reichler L., Munzinger M., Lin J. (2014, October 1). The impact of guidance on internet-based mental health interventions - a systematic review. Internet Interv..

[bb0035] Bostock S.K., Steptoe A. (2013). Can finding Headspace reduce work stress? A randomised controlled workplace trial of a mindfulness meditation app. Psychosom. Med..

[bb0040] Bower P., Kontopantelis E., Sutton A., Kendrick T., Richards D.A., Gilbody S., Liu E.T. (2013). Influence of initial severity of depression on effectiveness of low intensity interventions: meta-analysis of individual patient data. BMJ (Clinical Research Ed.).

[bb0045] Braun V., Clarke V. (2006). Using thematic analysis in psychology. Qual. Res. Psychol..

[bb0050] Brooke J. (2011). SUS - A Quick and Dirty Usability Scale. http://www.tbistafftraining.info/smartphones/documents/b5_during_the_trial_usability_scale_v1_09aug11.pdf.

[bb0055] Burcusa S.L., Iacono W.G. (2007). Risk for recurrence in depression. Clin. Psychol. Rev..

[bb0060] Cavanagh K., Strauss C., Forder L., Jones F. (2014). Can mindfulness and acceptance be learnt by self-help?: a systematic review and meta-analysis of mindfulness and acceptance-based self-help interventions. Clin. Psychol. Rev..

[bb0065] Craig P., Dieppe P., Macintyre S., Health P., Unit S., Michie S., Petticrew M. (2008). Developing and Evaluating Complex Interventions: New Guidance.

[bb0070] Craig P., Dieppe P., Macintyre S., Michie S., Nazareth I., Petticrew M. (2008). Developing and evaluating complex interventions: the new Medical Research Council guidance. BMJ (Clinical Research Ed.).

[bb0075] Crane C., Crane R.S., Eames C., Fennell M.J.V., Silverton S., Williams J.M.G., Barnhofer T. (2014). The effects of amount of home meditation practice in Mindfulness Based Cognitive Therapy on hazard of relapse to depression in the Staying Well after Depression Trial. Behav. Res. Ther..

[bb0080] Crane R.S., Kuyken W. (2012). The implementation of mindfulness-based cognitive therapy: learning from the UK Health service experience. Mindfulness.

[bb0085] Crawford M.J., Thana L., Farquharson L., Palmer L., Hancock E., Bassett P., Parry G.D. (2016). Patient experience of negative effects of psychological treatment: results of a national survey. Br. J. Psychiatry.

[bb0090] Cuijpers P., Marks I.M., van Straten A., Cavanagh K., Gega L., Andersson G. (2009). Computer-aided psychotherapy for anxiety disorders: a meta-analytic review. Cogn. Behav. Ther..

[bb0095] Devilly G.J., Borkovec T.D. (2000). Psychometric properties of the credibility/expectancy questionnaire. J. Behav. Ther. Exp. Psychiatry.

[bb0100] Duncan B.L., Miller S.D., Sparks J.A., Claud D.A., Reynolds L.R., Brown J., Johnson L.D. (2003). The session rating scale: preliminary psychometric properties of a “working” alliance measure. J. Brief Ther..

[bb6000] Elliott R., Slatick E., Urman M. (2001). Qualitative change process research on psychotherapy: alternative strategies. Psychol. Test Assess. Model..

[bb0105] Enrique A., Palacios J.E., Ryan H., Richards D. (2019). Exploring the relationship between usage and outcomes of an internet-based intervention for individuals with depressive symptoms: secondary analysis of data from a randomized controlled trial. J. Med. Internet Res..

[bb0110] Fish M.T., Saul A.D. (2019). The gamification of meditation: a randomized-controlled study of a prescribed mobile mindfulness meditation application in reducing college students’ depression. Simul. Gaming.

[bb0115] Fisher R.A. (1992). Statistical Methods for Research Workers.

[bb0120] Flett J.A.M., Hayne H., Riordan B.C., Thompson L.M., Conner T.S. (2019). Mobile mindfulness meditation: a randomised controlled trial of the effect of two popular apps on mental health. Mindfulness.

[bb0125] Goldberg S.B., Tucker R.P., Greene P.A., Davidson R.J., Wampold B.E., Kearney D.J., Simpson T.L. (2018). Mindfulness-based interventions for psychiatric disorders: a systematic review and meta-analysis. Clin. Psychol. Rev..

[bb0130] Gu J., Strauss C., Bond R., Cavanagh K. (2015). How do mindfulness-based cognitive therapy and mindfulness-based stress reduction improve mental health and wellbeing? A systematic review and meta-analysis of mediation studies. Clin. Psychol. Rev..

[bb0135] Gu Jenny, Strauss C., Crane C., Barnhofer T., Karl A., Cavanagh K., Kuyken W. (2016). Examining the factor structure of the 39-item and 15-item versions of the five-facet mindfulness questionnaire before and after mindfulness-based cognitive therapy for people with recurrent depression. Psychol. Assess..

[bb0140] Gu Jenny, Cavanagh K., Strauss C. (2018). Investigating the specific effects of an online mindfulness-based self-help intervention on stress and underlying mechanisms. Mindfulness.

[bb0145] Headspace (2020). Headspace Press and Media Webpage. https://www.headspace.com/press-and-media.

[bb0150] Headspace. (n.d.). Headspace. Retrieved from www.headspace.com.

[bb0155] Health and Social Care Information Centre (HSCIC) (2019). Psychological Therapies*,* Annual Report on the Use of IAPT Services - England. https://digital.nhs.uk/data-and-information/publications/statistical/psychological-therapies-annual-reports-on-the-use-of-iapt-services/annual-report-2018-19.

[bb0160] Jakobsen J.C., Gluud C., Wetterslev J., Winkel P. (2017). When and how should multiple imputation be used for handling missing data in randomised clinical trials - a practical guide with flowcharts. BMC Med. Res. Methodol..

[bb0165] Jayewardene W.P., Lohrmann D.K., Erbe R.G., Torabi M.R. (2017, March 1). Effects of preventive online mindfulness interventions on stress and mindfulness: a meta-analysis of randomized controlled trials. Prev. Med. Rep..

[bb0170] Kroenke K., Spitzer R.L., Williams J.B. (2001). The PHQ-9: validity of a brief depression severity measure. J. Gen. Intern. Med..

[bb0175] Kuyken W., Warren F.C., Taylor R.S., Whalley B., Crane C., Bondolfi G., Dalgleish T. (2016). Efficacy of mindfulness-based cognitive therapy in prevention of depressive relapse. JAMA Psychiat..

[bb0180] Lever Taylor B., Strauss C., Cavanagh K., Jones F. (2014). The effectiveness of self-help mindfulness-based cognitive therapy ina student sample: a randomised controlled trial. Behav. Res. Ther..

[bb0185] Ma S.H., Teasdale J.D. (2004). Mindfulness-based cognitive therapy for depression: replication and exploration of differential relapse prevention effects. J. Consult. Clin. Psychol..

[bb0190] McCrone P., Dhanasiri S., Patel A., Knapp M., Lawton-Smith S. (2008). Paying the Price: The Cost of Mental Health Care in England to 2026.

[bb0195] McManus S., Meltzer H., Brugha T.T., Bebbington P.P., Jenkins R. (2009). Adult Psychiatric Morbidity in England, 2007 Results of a Household Survey.

[bb0200] Meyer T.J., Miller M.L., Metzger R.L., Borkovec T.D. (1990). Development and validation of the penn state worry questionnaire. Behav. Res. Ther..

[bb0205] Mohr D.C., Cuijpers P., Lehman K. (2011, March 10). Supportive accountability: a model for providing human support to enhance adherence to eHealth interventions. J. Med. Internet Res..

[bb0210] National Institue of Health and Care Excellence [NICE] (2011). Common Mental Health Disorders.

[bb0215] National Institute for Health and Care Excellence [NICE] (2009). Depression in Adults With Chronic Physical Health Problems: Treatment and Management.

[bb0220] NHS Digital (2019). Psychological Therapies*,* Annual Report on the Use of IAPT Services 2018*-*19. https://digital.nhs.uk/data-and-information/publications/statistical/psychological-therapies-annual-reports-on-the-use-of-iapt-services/annual-report-2018-19.

[bb0225] Raes F., Pommier E., Neff K.D., Van Gucht D., Wiley J. (2011). Construction and factorial validation of a short form of the self-compassion scale.

[bb0230] Richards D., Timulak L., O’Brien E., Hayes C., Vigano N., Sharry J., Doherty G. (2015). A randomized controlled trial of an internet-delivered treatment: its potential as a low-intensity community intervention for adults with symptoms of depression. Behav. Res. Ther..

[bb0235] Richards Derek, Richardson T. (2012, June 1). Computer-based psychological treatments for depression: a systematic review and meta-analysis. Clin. Psychol. Rev..

[bb0240] Rycroft-Malone J., Gradinger F., Owen Griffiths H., Anderson R., Crane R.S., Gibson A., Kuyken W. (2019). “Mind the gaps”: the accessibility and implementation of an effective depression relapse prevention programme in UK NHS services: learning from mindfulness-based cognitive therapy through a mixedmethods study. BMJ Open.

[bb0245] Schueller S.M., Tomasino K.N., Mohr D.C. (2017, March 1). Integrating Human Support Into Behavioral Intervention Technologies: The Efficiency Model of Support.

[bb7000] Sheehan D.V. (2014). Mini International Neuropsychiatric Interview (MINI) for DSM 5 (Version 7.0.0).

[bb0255] Spijkerman M.P.J., Pots W.T.M., Bohlmeijer E.T. (2016, April 1). Effectiveness of online mindfulness-based interventions in improving mental health: a review and meta-analysis of randomised controlled trials. Clin. Psychol. Rev..

[bb0260] Spitzer R.L., Kroenke K., Williams J.B.W., Löwe B. (2006). A brief measure for assessing generalized anxiety disorder: the GAD-7. Arch. Intern. Med..

[bb0265] Sterne J.A.C., White I.R., Carlin J.B., Spratt M., Royston P., Kenward M.G., Carpenter J.R. (2009, July 18). Multiple imputation for missing data in epidemiological and clinical research: potential and pitfalls. BMJ (Online).

[bb0270] Stewart-Brown S., Tennant A., Tennant R., Platt S., Parkinson J., Weich S. (2009). Internal construct validity of the Warwick-Edinburgh Mental Well-being Scale (WEMWBS): a Rasch analysis using data from the Scottish Health Education Population Survey. Health Qual. Life Outcomes.

[bb0275] Strauss C., Cavanagh K., Oliver A., Pettman D. (2014). Mindfulness-based interventions for people diagnosed with a current episode of an anxiety or depressive disorder: a meta-analysis of randomised controlled trials. PLoS ONE.

[bb0280] Treynor W., Gonzalez R., Nolen-hoeksema S. (2003). Rumination Reconsidered: A Psychometric Analysis.

[bb0285] Victorson D.E., Sauer C.M., Wolters L., Maletich C., Lukoff K., Sufrin N. (2020, August 1). Meta-analysis of technology-enabled mindfulness-based programs for negative affect and mindful awareness. Mindfulness.

[bb0290] White I.R., Royston P., Wood A.M. (2011). Multiple imputation using chained equations: issues and guidance for practice. Stat. Med..

[bb0295] World Health Organization (2020). Depression. https://www.who.int/news-room/fact-sheets/detail/depression.

[bb0300] Zhang R., Nicholas J., Knapp A.A., Graham A.K., Gray E., Kwasny M.J., Mohr D.C. (2019). Clinically meaningful use of mental health apps and its effects on depression: mixed methods study. J. Med. Internet Res..

